# The presence of partial compaction patterns is associated with lower rates of blastocyst formation, sub-optimal morphokinetic parameters and poorer morphologic grade

**DOI:** 10.1186/s12958-023-01059-9

**Published:** 2023-01-28

**Authors:** Christine Hur, Vaani Nanavaty, Meng Yao, Nina Desai

**Affiliations:** 1 Department of Obstetrics and Gynecology, Women’s Health Institute, Cleveland Clinic, Division of Reproductive Endocrinology and Infertility, 26900 Cedar Road, Beachwood, OH 44122 USA; 2 Quantitative Health Sciences, Cleveland Clinic, 9500 Euclid Ave. JJN3, Cleveland, OH 44195 USA

**Keywords:** Embryo compaction, Time-lapse imaging, Morphokinetics, Pre-implantation genetic testing

## Abstract

**Background:**

Compaction is an important marker of embryonic genome activation and marks a critical step in the development to blastocyst. The objective of our study was to determine whether visualization of the embryonic compaction process through time-lapse imaging (TL) can assist in predicting the kinetics of embryo development as well as the likelihood for blastocyst formation, grade, or ploidy.

**Methods:**

This study is a retrospective review of prospectively collected data from a single academic institution. Couples included were those who underwent preimplantation genetic testing for aneuploidy (PGT-A) following in vitro fertilization between January and December 2020. Embryos were cultured in the Embrysocope. Embryo morphokinetic data was prospectively collected and analyzed. TL videos were later reviewed in detail for compaction pattern. Embryo compaction patterns (CP) were categorized as follows: 1) full compaction (CP-F), 2) partial compaction with cell extrusion (P-ext), 3) partial compaction with cell exclusion (P-exc) and 4) partial compaction with both cell extrusion and exclusion (P-both). Assessment of embryo decompaction and re-compaction was evaluated. The association between CP, morphokinetic parameters, blastocyst formation, grade and ploidy were then analyzed.

**Results:**

A total of 349 embryos were studied. Amongst embryos which progressed to morula (*n* = 281), the distribution of compaction patterns were: CP-F 45.6%, P-ext 12.5%, P-exc 29.5% and P-both 12.5%. Embryos exhibiting a CP-F were more likely to proceed to blastocyst compared with those that demonstrated partial compaction patterns (*p* = 0.006). When compared to CP-F, partial compaction patterns were significantly associated with poorer ICM and TE grades (*P* < 0.001). Of the 281 morula, 59.8% (*n* = 168) demonstrated at least one episode of decompaction and re-compaction. Of the 249 blastocysts formed, 200 were cryopreserved for future use after undergoing PGT-A evaluation. Of those, 42.5% were diagnosed as euploid, 39.0% as aneuploid, 9.0% as mosaic and 9.5% had no result. When compared to CP-F, partial CPs exhibited a significantly greater percentage of mosaic embryos (3.6% v. 15.6%, *p* = 0.032). Additionally, we found that a greater percentage of embryos demonstrating CP-F exhibited morphokinetics that fell into optimal ranges for embryo development when compared to those with partial compaction patterns.

**Conclusion:**

Time-lapse visualization of compaction patterns identified exclusions and/or extrusions as negative indicators of blastocyst formation and blastocyst grade. When compared to full compaction patterns, partial compaction patterns were associated with delayed embryonic development as well as lower rates of optimal kinetic development.

## Background

The earliest stages of embryo development are largely under the molecular control of maternally inherited mRNAs and regulatory proteins stored throughout oogenesis. This initial maternal control is gradually superseded as the embryo expresses its own genes with the activation of the embryonic genome. Embryonic genome activation (EGA) in the human embryo is believed to occur at the 4 to 8 cell stage [1]. Prior to compaction, the cleavage stage embryo is comprised of relatively equivalent and independent blastomeres [2]. The individual blastomeres then flatten their membranes against one another, rapidly increasing cell-to-cell contactdue to an increase in adhesion protein expression [3]. This increased cell to cell adherence leading to compaction is the earliest outward indicator of embryonic gene expression.

Subsequently, a growing number of cell junctions develop, increasing intercellular communication between the individual blastomeres [4]. This allows the embryo to develop into a cohesive mass of cells known as a morula, defined by tightly adherent blastomeres with ill-defined margins no longer distinguishable from one another. Following this, the preimplantation embryo is found to be metabolically, molecularly and cellularly different [5–8]. Therefore, compaction marks a critical transition point in human embryo development.

As IVF labs have moved towards single embryo transfers (SETs), embryologists strive to improve embryo selection to maintain favorable pregnancy rates. Currently, the primary methods for embryo selection include embryo morphology, preimplantation genetic testing for aneuploidy (PGT-A), as well as embryo morphokinetics. Using these methods, IVF success rates have improved over the past decades, but embryologists and reproductive endocrinologists continue to observe failed embryo transfers, even with euploid embryos with reassuring morphology and morphokinetics. This suggests that there continues to be more to learn regarding embryo growth and viability.

Despite the significance of compaction in early embryo development, patterns of compaction and morula development have been poorly researched. By further investigating the compaction process, we may better understand early embryo development and the impact it has on later clinical outcomes. Compaction may take hours to complete and the window at which this process is initiated is also variable. This dynamic nature of the compaction process and the constraints imposed by once daily observations in conventional in vitro fertilization (IVF) culture systems have likely contributed to compaction being understudied. Amongst the studies performed, most have relied on once-daily morphology to describe embryo compaction [9–14]. More recently, time-lapse microscopy (TLM) has been utilized to evaluate compaction [15–17].

In the present investigation, we make a detailed analysis of the entire compaction process using time lapse imaging. The objective of our study was to determine whether this detailed visualization of the embryonic compaction process can assist in predicting the likelihood for blastocyst formation, timing, morphologic grade, ploidy and subsequent clinical outcomes.

## Methods

### Study design and patient cohort

This study conducts a retrospective analysis of prospectively collected morphokinetic and PGT-A data for 30 couples undergoing 37 cycles of in vitro fertilization with intracytoplasmic sperm injection (ICSI) cycles. Embryos which underwent preimplantation genetic testing for aneuploidies (PGT-A) analysis from January to December of 2020 were included. PGT-A was elective and offered to all patients, but recommended for women 38 years and older. Couples with all diagnoses and stimulation protocols were included. This study was conducted under appropriate IRB approval (IRB# IVF data registry IRB#5251 and EmbryoScope data registry IRB# 14–566) and within the guidelines provided by the Cleveland Clinic Institutional Review Board.

### Compaction patterns

Compaction was defined as the loss of visualization of cell boundaries between three or more neighboring blastomeres. The compaction interval ranged from tSC (start of compaction) to tSB (start of blastulation). Compaction patterns were divided into four categories as follows: full compaction (CP-F), partial compaction with cell exclusion (P-exc), partial compaction with cell extrusion (P-ext) and partial compaction with both cell exclusion and extrusion (P-both). Care was taken to distinguish between large fragments and blastomeres. Extruded or excluded blastomeres were identified based on presence of a nucleus or on relative size compared to other blastomeres at that stage of development.

**Fig. 1:**
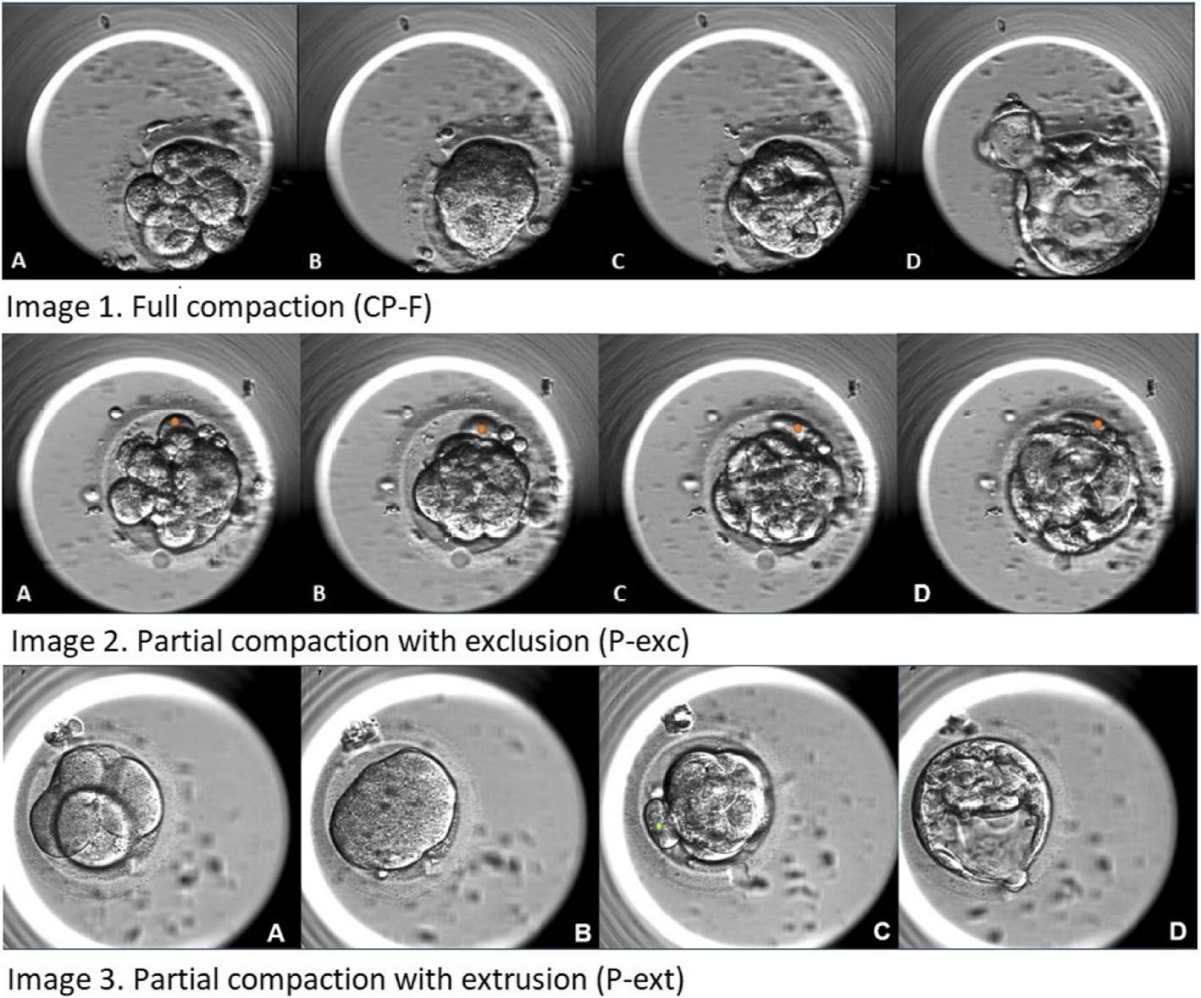
Early embryo development and compaction patterns. Three primary compaction patterns were identified and a fourth categorization was made for embryos that exhibit both cell extrusion and exclusion. 1) CP-F, fully compacted morula; 2) P-exc, partially compacted morula with blastomere excluded from morula development, 3) P-ext, partially compacted morula with blastomere excluded following p*revious integration into morula*

Compaction patterns were defined as follows (Fig. 1):

1. Full compaction (CP-F) describes the morula stage embryo, when all the individual blastomeres (image 1, panel A) are included in the compaction process and form a single cellular mass (image 1, panel B) and subsequently develop into the blastocyst (image 1, panel C and D).
2. Partial compaction withcell exclusion (P-exc) describes a partial compaction pattern wherein one or more of the blastomeres are not included in the compaction process for the duration of embryo development (image 2). Therefore, one or more blastomere(s) are not included into the morula (image 2, panel B) or subsequently into blastocyst development (image 2, panel D). The blastomere(s) remain distinct and independent throughout early embryo development.
3. Partial compaction with cell extrusion (P-ext) describes when one or more blastomere(s) are initially included in the compaction process, but then subsequently become independent again prior to the development of the blastocyst. Although a blastomere is initially integrated into the morula (image 3, panel B), ultimately it is not included into the development of the blastocyst (image 3, panel D). Extruded blastomeres were included for analysis if they were extruded prior to the start of tSB.
4. Partial compaction with both cell exclusion and extrusion (P-both) describes a partial compaction pattern where blastomeres have been both excluded and extruded during the compaction process.

The number of blastomeres present prior to compaction were categorized into three groups for analysis: A) six or fewer, B) seven to ten and C) greater than eleven. In addition, episodes of decompaction and re-compaction were quantified. Decompaction was defined as the visualization of independent blastomere(s) following anearlier compaction event. Re-compaction describes the reintegration of these blastomere(s) into the morula. The presence of episodes of decompactions and compactions were noted. We also evaluated if there were multiple occurrences and if there was an association with continuing cell division.

Compaction patterns of all embryos were visualized by a single observer. A second observer visualized 20% of embryos for quality control. Any discrepancies between the two observers were reviewed by a third observer, the IVF lab director as needed to clarify categorization of the compaction pattern.

### Ovarian stimulation

Stimulation protocols were selected based on maternal age, serum anti-müllerian hormone (AMH) level, antral follicle count (AFC) and prior ovarian stimulation response when applicable. Ovarian stimulation was performed with standard controlled ovarian stimulation protocols. Once two lead follicles of at least 18 mm in size were formed, final follicular maturation was triggered with an injection of GnRH agonist or hCG. Ultrasound-guided follicular aspiration was performed 36 hours following trigger.

### Fertilization and embryo culture

After oocyte retrieval, cumulus-oocyte complexes (COCs) were incubated for 2–3 hours in HTF medium (GMHT-100; Life Global, Connecticut, USA) supplemented with 10% human serum albumin (ART-3003; Cooper-Surgical, Connecticut, USA) with an oil overlay at 37 °C with 6% CO_2_ and air. Cumulus cells surrounding oocytes were removed using enzymatic digestion with hyaluronidase (ART-4007-A; Cooper-Surgical, Connecticut, USA). ICSI was then performed on mature (metaphase II) oocytes while immature oocytes were co-incubated with sperm. At 16–18 hours post insemination, fertilization was assessed. Normally fertilized pronuclear embryos were transferred to individual wells of an EmbryoSlide, containing pre-equilibrated medium with an overlay of oil [18]. Embryos were cultured at 37 °C with 6% CO_2_ and 6% O_2_ until the blastocyst stage.

### Embryo morphokinetics

The EmbryoScope Time-Lapse System (Vitrolife) was used to monitor embryo development, providing high-definition images of each embryo every 15 minutes at 5–7 focal planes. Embryo development was assessed by viewing of time-lapse videos and annotating for cell division as well as morphology.

Timing to 2-cell (t2), t3, t4, t5, t8, compaction (tSC), morula (tM), start of blastocyst (tSB), blastocyst (tB), expanded blastocyst (tEB) and hatching blastocyst (tHB) were annotated. The start of ICSI was considered t0 and all time-points were expressed as hours post-insemination (hpi). Timing of specific cell cycle events such as cc2 (t3-t2), s2 (t4-t3) and cc3 (t5-t3) were calculated. tSC was defined as the time point at which the embryo showed increase cell to cell adherence with three or more merged blastomeres, with loss of individual cell borders. tM was the time point at which over 90% of blastomeres were merged. Given that all embryos were hatched on day 3 to facilitate trophectoderm biopsy, zona thinning and increase in diameter could not be used as a marker for timing of blastocyst expansion. Instead, we used the herniation of 3–4 trophectodermal cells from the opening in the zona as an indicator of blastocyst expansion (tEB) and tHB was defined as the point at which 5 or more trophectodermalcells were observed outside of the zona.

Embryos were further assessed to determine whether they fell into optimal kinetic ranges for cell cycle parameters established in prior published work [19–24]. Established optimal ranges are defined as follows: cc2 (> 5 and ≤ 11.9 h), s2 (≤ 1 h), t5 (45–57 h), cc3 (9.7–21 h), tSB (< 96.2 h) and tEB (≤ 116 h).

### Blastocyst grading

Morphology was graded by blastocyst expansion, inner cell mass (ICM) morphology and trophectoderm (TE) grade using the ESHRE/Alpha scoring [25]. Blastocyst expansion scoring was as follows: 1 for early, 2 for full, 3 for expanded and 4 for hatched. ICM morphology was scored as 1 for good, 2 for fair and 3 for poor or absent. TE grade is based on overall organization and number with a score of 1 for good, 2 for fair and 3 for poor.

### Preimplantation genetic testing for aneuploidy (PGT-A)

On day 3 of development, a small opening was made in the zona pellucida of each embryo using a Hamilton-Throne Lykos laser. This opening facilitated the herniation of trophectoderm cells on day 5 or 6 for biopsy. TE biopsy was performed on expanded and hatched blastocysts with removal of 5–8 cells. Biopsied cells were then sent to a commercial laboratory (iGenomix) for analysis. PGT-A testing was performed using next generation sequencing of all 24 chromosomes. Blastocysts were reported as being euploid, aneuploidy, mosaic or no result based on the analysis of biopsied cells.

### Statistical analysis

Approximately normally-distributed continuous measures were summarized using means and standard deviations and compared using two-sample t-tests or ANOVA. Continuous measures that show departure from normality were compared using Wilcoxon rank sum tests or Kruskal-Wallis tests. Categorical factors were summarized using frequencies and percentages and were compared using Pearson’s chi-square tests or Fisher’s exact tests. Post-hoc pairwise comparisons were done using Bonferroni adjustment. All analyses were done using SAS (version 9.4, The SAS Institute, Cary, NC) and a *p* < 0.05 was considered statistically significant.

## Results

### Compaction patterns

We studied the compaction patterns of 349 embryos from 30 couples undergoing 37 independent IVF/ICSI cycles. Patient demographics and cycle characteristics are demonstrated in Table 1. Of these, 281 (80.5%) progressed to morula. The compaction patterns amongst these 281 embryos were assessed (Table 2). Just underhalf of the embryos demonstrated CP-F (*n* = 128, 45.6%), while approximately a third of the embryos demonstrated P-exc (*n* = 83, 29.5%). P-ext(*n* = 35, 12.5%) and P-both (*n* = 35, 12.5%) each represented one eighth of the cohort. Of embryos which exhibited a P-ext compaction pattern, the majority extruded two or fewer blastomeres. Embryos with a P-exc pattern demonstrated a wider range of cells excluded. Most embryos (68.7%) had 11 or more blastomeres present prior to compaction. Approximately a third of embryos underwent compaction with 10 or fewer blastomeres. When comparing CP-F with P-ext and P-exc patterns, a significant association was seen between the number of blastomeres prior to compaction and compaction pattern with a greater number of embryos undergoing full compaction having ≥11 blastomeres prior to compaction (*p* = 0.004) (Table 3).

**Table 1:** Patient characteristics

	***n*** = 37
Age (maternal)	36.5 ± 4.2
Gravidity	1.00 [0.00, 2.0]
Parity	0.00 [0.00, 1.00]
AMH	2.1 ± 1.5
BMI	25.9 ± 5.1
Tobacco use	
No	28 (75.7)
Former	9 (24.3)
Prior IVF cycles	
0	24 (64.9)
1	11 (29.7)
2+	2 (5.4)

Statistics presented as Mean ± SD, Median [P25, P75], n (%)

**Table 2:** Embryo development and compaction patterns

	n	
Embryo outcome	349	
Arrested prior to morula		68 (19.5)
Achieved morula		281 (80.5)
Blastocyst		249 (71.3)
Blastocyst, PGT-A biopsy		200 (57.3)
Compaction Pattern	281	
CP-F		128 (45.6)
Partial compaction		153 (54.4)
P-extruded (P-ext)		35 (12.5)
P-excluded (P-exc)		83 (29.5)
P-both		35 (12.5)
Extruded cells	153	
0		83 (54.2)
1		37 (24.2)
2		18 (11.8)
3		11 (7.2)
4		3 (2.0)
5+		1 (0.65)
Excluded cells	153	
0		35 (22.9)
1		41 (26.8)
2		18 (11.8)
3		13 (8.5)
4		16 (10.5)
5+		30 (19.6)

Statistics presented as n (%)

**Table 3:** Comparison of number of blastomeres prior to compaction by compaction pattern

	CP-Full ***n*** = 128	P-Extruded ***n*** = 35	P-Excluded ***n*** = 83	P-Both ***n*** = 35	
# of Blastomeres					***p*-value**
≤ 6	3 (2.3) ^2^	6 (17.1) ^1,3^	0 (0.00) ^2^	0 (0.00)	***0.004***^***b***^
7–10	31 (24.2)	13 (37.1)	24 (28.9)	11 (31.4)	
≥ 11	94 (73.4)	16 (45.7)	59 (71.1)	24 (68.6)	

Statistics presented as n (%).*p*-value < 0.05 is considered statistically significant. ^b^Kruskal-Wallis test

^1^: Significantly different from CP-F; ^2^Significantly different from P-extruded; ^3^Significantly different from P-Excluded; ^4^Significantly different from P-Both. Post-hoc pairwise comparisons were done using Bonferroni adjustment

We also examined the relationship between CP patterns, decompaction, re-compaction and cell division as shown in Table 4. Of the 281 morula, 59.8% (*n* = 168) demonstrated decompaction and subsequent re-compaction. Of these, 82.2% embryos had only one episode of decompaction and re-compaction while the minority (17.8%) demonstrated multiple episodes. Amongst the 168 embryos exhibiting at least one episode of decompaction with re-compaction, 28.5% had decompaction associated with further cell division. A significantly lower rate of decompaction was observed with the P-exc pattern as compared to embryos which exhibited either a CP-F or P-ext pattern (*p* < 0.05 after Bonferroni adjustment). Decompaction amongst embryos in the P-extgroup was found to have a higher association with cell division compared to other partial compaction patterns (*p* < 0.001). No association was seen between the number of decompaction events and compaction pattern.

**Table 4:** Comparison of the presence of decompaction and re-compaction by compaction pattern

	CP-Full ***n*** = 128	P-Extruded ***n*** = 35	P-Excluded ***n*** = 83	P-Both ***n*** = 35	***p***-value
Presence of decompaction/re-compaction episode(s)					***0.007***^***c***^
No	43 (33.6) ^3^	9 (25.7) ^3^	44 (53.0) ^12^	17 (48.6)	
Yes	85 (66.4)	26 (74.3)	39 (47.0)	18 (51.4)	
Assocociated w/ cell division					***< 0.001***^***c***^
No	86 (67.2)	16 (45.7) ^34^	69 (83.1) ^2^	30 (85.7) ^2^	
Yes	42 (32.8)	19 (54.3)	14 (16.9)	5 (14.3)	
Multiple decompaction episodes					0.056^c^
No	98 (76.6)	28 (80.0)	72 (86.7)	33 (94.3)	
Yes	30 (23.4)	7 (20.0)	11 (13.3)	2 (5.7)	

Statistics presented as n (%). *p*-value < 0.05 is considered statistically significant. ^c^Pearson’s chi-square test

^1^Significantly different from CP-F; ^2^Significantly different from P-extruded; ^3^Significantly different from P-Excluded; ^4^Significantly different from P-Both. Post-hoc pairwise comparisons were done using Bonferroni adjustment

### Blastocyst formation, grade and ploidy

Of the 349 embryos cultured, 281 progressed to morula and were further analyzed. The overall blastulation rate was 71% (249/349). A total of 200 high-quality blastocysts underwent trophectoderm biopsy for PGT-A testing and were then cryopreserved (Table 2). For embryos which exhibited a CP-F pattern, 59.1% were biopsied and frozen on day 5 of embryo development, while the rates were significantly lower for the P-ext, P-exc, and P-both (22.7, 34.0 and 33.0%; *p* < 0.001). Additionally, a significant difference in rates of blastocyst formation was identified between CP-F (95.3%) and partial compaction patterns: P-ext (82.9%), P-exc (83.1%) and P-Both (82.9%) (*p* = 0.006) (Table 5).

**Table 5:** Comparison of blastocyst formation and biopsy date by compaction pattern

	CP-F (***n*** = 128)		P-extruded (***n*** = 35)		P-Excluded (***n*** = 83)		P-Both (***n*** = 35)		
	n		n		n		n		***p***-value
Blastocyst formation	128		35		83		35		***0.006***^***d***^
No		6 (4.7) ^3^		6 (17.1)		14 (16.9) ^1^		6 (17.1)	
Yes		122 (95.3)		29 (82.9)		69 (83.1)		29 (82.9)	
Day of biopsy	110		22		47		21		***< 0.001***^***c***^
5		65 (59.1) ^23^		5 (22.7) ^1^		16 (34.0) ^1^		7 (33.3)	
6		45 (40.9)		17 (77.3)		31 (66.0)		14 (66.7)	

Statistics presented as n (%). *p*-value < 0.05 is considered statistically significant. ^c^Pearson’s chi-square test, ^d^Fisher’s Exact test

^1^Significantly different from CP-F; ^2^Significantly different from P-extruded; ^3^Significantly different from P-Excluded; ^4^Significantly different from P-Both. Post-hoc pairwise comparisons were done using Bonferroni adjustment

When comparing full compaction patterns with all partial compaction patterns as a pooled group (Table 6), significant differences were detected in blastocyst maturity (*p* = 0.029), ICM morphology (*p* < 0.001) as well as TE grade (*p* < 0.001). Individual partial compaction patterns, when compared to CP-F showed significantly lower ICM (*p* < 0.001) and TE grades (*p* < 0.001). We also examined relationship between compaction patterns and two specific embryo dysmorphisms, irregular chaotic division (ICD) and multinucleation. ICD was significantly associated with partial compaction (CP 7.8%, P-Ext 5.7%, P-Exc 22.9%, P-Both 22.9%; *p* = 0.0031). No difference in rate of multinucleation was identified between the different compaction patterns (CP 52.3%, P-Ext 57.1%, P-Exc 45.8%, P-Both 37.1%).

**Table 6:** Comparison of blastocyst grade and ploidy by compaction patterns between CP-F and pooled partial compaction patterns (P-ext, P-exc, P-both)

		CP-F (***N*** = 128)		Pooled Partial Patterns (***N*** = 153)		
	Total (***n*** = 281)	n		n		***p***-value
Blastocyst Maturity		122		128		***0.029***^***b***^
4	181 (72.4)		95 (77.9)		86 (67.2)	
3	48 (19.2)		23 (18.9)		25 (19.5)	
2	20 (8.0)		4 (3.3)		16 (12.5)	
1	1 (0.40)		0 (0.00)		1 (0.78)	
Inner Cell Mass Morphology		122		128		***< 0.001***^***b***^
1	93 (37.2)		60 (49.2)		33 (25.8)	
2	100 (40.0)		54 (44.3)		46 (35.9)	
3	57 (22.8)		8 (6.6)		49 (38.3)	
Trophectoderm Grade		122		128		***< 0.001***^***b***^
1	88 (35.2)		54 (44.3)		34 (26.6)	
2	105 (42.0)		54 (44.3)		51 (39.8)	
3	57 (22.8)		14 (11.5)		43 (33.6)	
Ploidy		110		90		***0.032***^***c***^
Euploid	85 (42.5)		51 (46.4)		34 (37.8)	
Aneuploid	78 (39.0)		44 (40.0)		34 (37.8)	
Mosaic	18 (9.0)		4 (3.6)		14 (15.6)	
No Result	19 (9.5)		11 (10.0)		8 (8.9)	

Statistics presented as n (%). *p*-value < 0.05 is considered statistically significant. ^b^Wilcoxon Rank Sum test, ^c^Pearson’s chi-square test

All 200 embryos meeting criteria for future transfer underwent PGT-A. Of these, 42.5% embryos were diagnosed as euploid, 39.0% as aneuploid, 9.0% as mosaic, and 9.5% had no result. When comparing CP-F versus pooled partial compaction patterns, a significant association was identified compaction pattern and ploidy with a significantly greater number of embryos that underwent partial compaction being identified as mosaic (15.6% v. 3.6%, *p* = 0.032) (Table 6).

### Timing of embryo development

When compared to CP-F, embryos with partial compaction patterns demonstrated delays in embryo development at each morphokinetics parameter (Table 7). For the start of compaction (tSC), CP-F embryos underwent compaction at 79.9 h on average, hours earlier than embryos which exhibited P-ext, P-exc and P-both (81.8, 88.4 and 85.5 h, respectively; *p* < 0.001). Similarly, tSB and tB also occurred hours earlier for CP-F embryos compared to those with partial compaction patterns (*p* < 0.001). Consistent with this, embryos exhibiting CP-F were biopsied on day 5 at nearly twice the rate of those with partial compaction patterns (*p* < 0.001) (Table 5).

**Table 7:** Comparison of morphokinetic parameters by compaction pattern

	CP-Full ***n*** = 128	P-Extruded ***n*** = 35	P-Excluded ***n*** = 83	P-Both ***n*** = 35	
***Timing Variables***					***p*-value**
t2	27.0 ± 2.4	28.6 ± 3.1	28.2 ± 4.0	27.8 ± 2.8	***0.012***^***a***^
t3	37.8 ± 3.7	40.8 ± 3.5	36.7 ± 5.6	36.6 ± 5.7	***< 0.001***^***a***^
t4	38.8 ± 3.6	42.2 ± 4.4	39.9 ± 5.5	40.7 ± 6.4	***0.001***^***a***^
t5	50.3 ± 6.3	52.2 ± 6.1	47.5 ± 9.2	47.8 ± 11.0	***0.008***^***a***^
t8	57.3 ± 8.6	63.7 ± 10.5	62.3 ± 10.8	61.9 ± 11.2	***< 0.001***^***a***^
tsc	78.9 ± 13.1	81.8 ± 13.8	88.4 ± 11.4	85.0 ± 12.9	***< 0.001***^***a***^
tm	84.8 ± 13.9	89.4 ± 13.7	96.5 ± 12.1	93.8 ± 15.3	***< 0.001***^***a***^
tsb	95.1 ± 15.4	104.9 ± 10.6	102.3 ± 12.4	102.0 ± 15.2	***< 0.001***^***a***^
tb	101.1 ± 16.2	110.9 ± 8.7	109.2 ± 13.3	110.0 ± 19.0	***< 0.001***^***b***^
teb	109.2 ± 19.5	125.2 ± 11.7	118.1 ± 14.8	116.1 ± 19.9	***< 0.001***^***b***^
thb	111.8 ± 21.6	128.1 ± 11.2	121.9 ± 16.2	120.2 ± 24.0	***< 0.001***^***b***^
cc2	10.8 ± 2.9	12.2 ± 2.2	8.5 ± 4.6	8.7 ± 5.2	***< 0.001***^***b***^
s2	0.94 ± 1.5	1.5 ± 2.4	3.1 ± 4.3	4.1 ± 6.2	***0.010***^***b***^
cc3	12.5 ± 3.6	11.4 ± 5.4	10.8 ± 7.0	11.2 ± 8.5	0.094^b^

Statistics presented as Mean ± SD. *p*-value < 0.05 is considered statistically significant. ^a^ANOVA, ^b^Kruskal-Wallis test

**Fig. 2:**
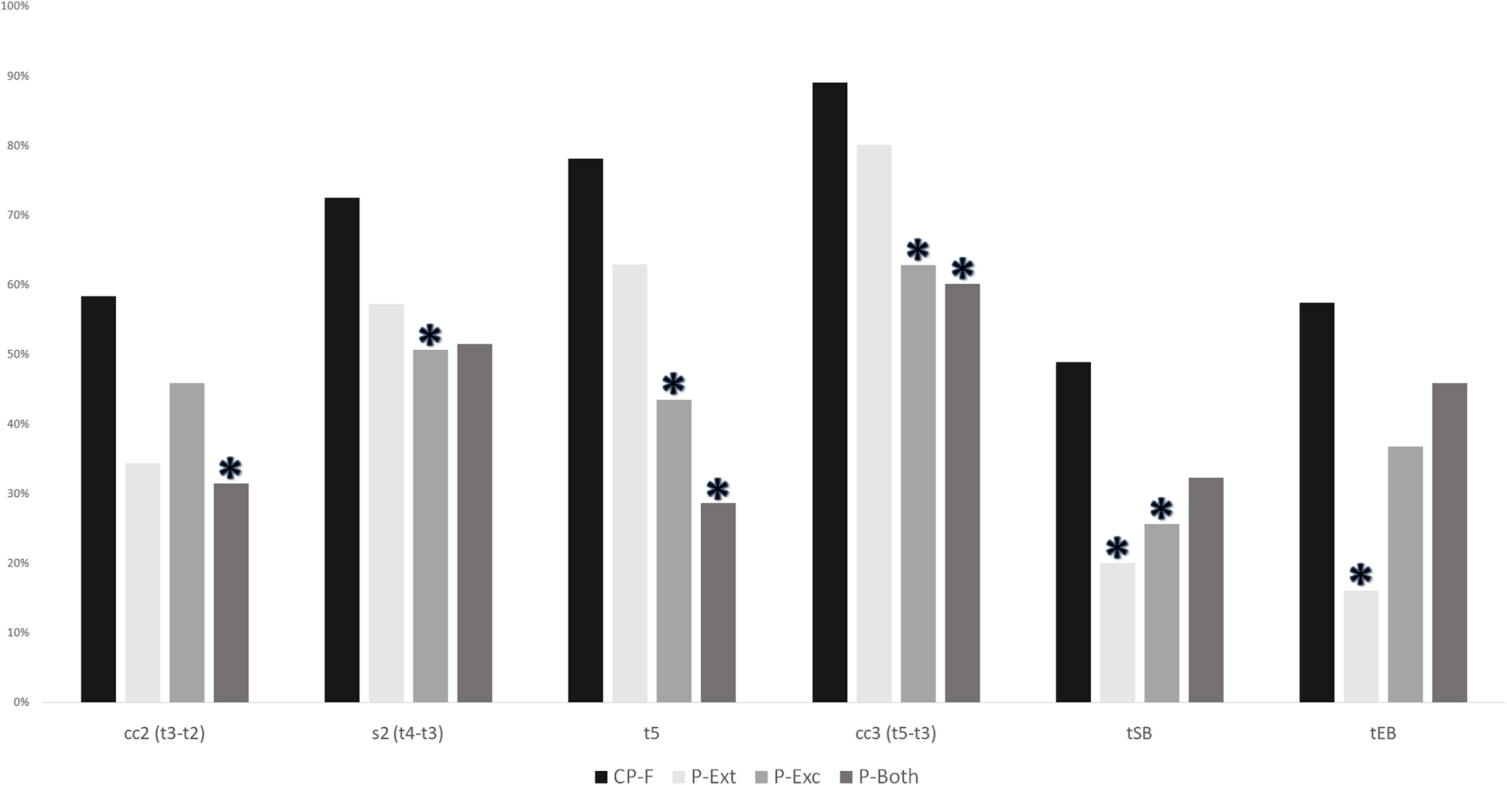
Compaction pattern and proportion of embryos with optimal kinetics of development. This graph shows the percentage of embryos with kinetic timings falling into optimal ranges. Optimal ranges were defined as follows: cc2 (> 5 and ≤ 11.9 h), s2 (≤ 1 h), t5 (45–57 h), cc3 (9.7–21 h), tSB (< 96.2 h) and tEB (≤ 116 h). *p* < 0.05 was considered statistically significant. (*) marks partial compaction patterns significantly different from CP-F

To further characterize embryo development, we determined the proportion of embryos that demonstrated optimal timing of embryo development to cc2, S2, cc3, t5, tSB, and tEB as shown in Fig. 2. This graph shows that a significantly greater percentage of embryos demonstrating CP-F fell within optimal timing when compared to those with partial compaction patterns (P-ext, P-exc, P-both).

### Clinical data

A total of 25 single embryo transfers have been performed to date. Amongst the transferred embryos, the distribution of compaction patterns was as follows: CP-F (*n* = 14), P-ext (*n* = 1), P-exc (*n* = 6) and P-Both (*n* = 4). A total of 17 clinical pregnancies were documented by early pregnancy ultrasound based on presence of a fetal pole with cardiac activity. The majority of embryos selected for transfer had the CP-F compaction pattern and the clinical pregnancy and live birth rates were 64.3% (9/14) and 57.1% (8/14), respectively. Clinical pregnancies and live births for transferred euploid blastocysts arising from embryos displaying partial compaction patterns was as follows: P-exc (5/6 and 4/6, respectively) and P-both (3/4 and 3/4, respectively).

## Discussion

Compaction occurs in a continuum over hours and sometimes days. Our present study describes different patterns of compaction, their prevalence and the clinical consequences. Our work looked in detail at the steps leading up to compaction and the subsequent compaction process. To date the majority of published data on human embryo compaction are based on single observations at set time intervals [9, 11–14]. Tao et al. proposed a four tier grading system for day 4 morula/compacting embryos based on degree and pattern of compaction. Others have simply classified compacting embryos as either fully or partially compacted [14]. Another approach for day 4 grading has been to score degree of compaction as well as fragmentation in day 4 embryos [12, 13]. The above-named studies provide evidence to a positive correlation between full compaction and blastocyst developmental potential as well as implantation potential using static observations. Only three studies besides our present work have used time lapse technology to better understand compaction [15, 17, 26]. Rienzi et al. reported that the live birth rate after transfer of a euploid blastocyst was predicted by time of morulation (tM) and quality of the trophectoderm [15]. Lagalla et al. classified partial embryo compaction patterns of PGT-A embryos in terms of whether cells were excluded or extruded during the compaction event [16].

Cell number in compacting embryos is widely variable. Early compaction of embryos at 6 or less cells has generally been viewed negatively within our lab. Using the analytical capability offered by TL imaging we were able to analyze this more objectively and confirm this observation. A very low percentage of fully compacted morula (2.3%) exhibited this behavior but it was observed in 17% of partially compacted CP-extembryos. Differences in average blastomere number at outset of compaction were also reported by Coticchio et al. between fully compacted morula (12.1 cells), partial CP-exc (11.9 cells) and partial CP-ext (11.7 cells) [17]. Early compaction was not reported on.

Another feature of morulation that has not received much attention is decompaction. The frequency of embryo decompaction events was high even amongst CP-F embryos and did not appear to be predominantly associated with cell division. The molecular basis and causes of embryonic decompaction are not clear. In our lab, we have certainly observed this as a precursor to cell degeneration in poor quality compacting embryos.

This pilot study utilizing TLM highlights the prevalence and variations in embryo compaction. Over half of the embryos studied exhibited a partial compaction pattern (54.4%). Other investigators classifying compaction patterns have reported partial compaction in only 36.2% of the embryo cohort [11]. This may represent a limitation of static morphology observations when describing a dynamic process. Moreover, episodes of decompaction and re-compaction were observed to occur in 59.8% of compacting embryos. Without TLM, dynamic findings such as transient decompaction and re-compaction would be missed. Morphokinetic benchmarks and optimal kinetic ranges have been identified that are associated with blastocyst development, implantation potential and ploidy [18–21, 23, 24]. These benchmarks when applied to embryos in this study revealed distinct differences based on compaction pattern. A significantly greater proportion of CP-Fembryos exhibited optimal timings for each cleavage event and major developmental benchmarks such as morulation, start of blastulation and expanded blastocyst formation. In contrast, embryos with a partial compaction pattern demonstrated delays in development and were significantly less likely to develop into blastocysts.

The etiology behind the different partial compaction patterns observed in human in vitro culture is still unknown. Possible explanations for the partial compaction phenotype include abnormalities in forming tight junctions, inability to express proteins required for cell-to-cell adhesion, or as a form of “aneuploid rescue” [8, 26, 27]. The theory of aneuploid rescue suggests the possibility that chromosomally abnormal blastomeres are being excluded in embryonic development. To examine this idea, Lagalla et al. performed chromosomal analysis on the excluded/extruded blastomeres of 18 embryos that exhibited apartial compaction pattern. They found that a disproportionate number of the excluded cells were aneuploid (38.5%) or degraded (46.2%) with the significant minority being euploid (15.4%). Yettrophectoderm biopsy of the resultant blastocysts revealed 72% of the embryosbe euploid. Moreover, in the blastocysts diagnosed as aneuploid, all the excluded/extruded cells were aneuploid [26]. Our datain directly supports this hypothesis. Mosaic embryos were significantly more likely to demonstrate partial compaction patterns than full compaction (15.6% v. 3.6%, *p*-value = 0.032). Another consideration is irregular cell divisions in the early cleavage embryo and its impact on subsequent cellular events, including compaction pattern. As described in these data, embryos which demonstrate cell exclusion have higher rates of irregular and chaotic division. This may be explained by cells that undergo irregular and chaotic divisions may end up fragmenting, becoming excluded during compaction, or end up in the TE and/or ICM, resulting in a mosaic embryo. Although our data does not demonstrate a significant difference in multinucleation between compaction patterns, this should continue to be investigated in larger studies.

The male genetic contribution to compaction pattern needs also to be considered. Given that compaction signifies the activation of the embryonic genome, and therefore expression of paternal inheritance, it would be interesting to determine to what degree the diagnosis of male infertility and its severity might influence embryonic compaction patterns. Two studies demonstrated that embryos from surgically extracted sperm due to severe male factor infertility are significantly delayed in achieving compaction when compared to embryos from ejaculated spermatozoa [28, 29]. Further, Desai et al. found that testicular-sperm derived embryos in cases of non-obstructive azoospermia were significantly less likely to undergo compaction at all. One explanation for this is that a disruption to spermatogenesis may potentially alter paternal gene expression [30]. Further study is needed to understand the potential influence of male infertility, sperm source and other clinical diagnoses on embryonic compaction.

A question of paramount interest is the implantation potential of blastocysts derived from partial compaction patterns. To our knowledge, only two studies have looked at clinical outcomes based on compaction pattern and they obtained conflicting results. The first study showed no difference in implantation or live birth rate [16]. In contrast a subsequent larger study by Coticchio and colleagues, reported partial compaction pattern to be associated with lower clinical pregnancy, ongoing pregnancy and live birth rates [17]. In the present work, we found that the majority of embryos selected for transfer demonstrated a complete compaction pattern. Although in this data, a clear trend in clinical pregnancy rate and live birth rate cannot be identified, this is due to our sample size and the small number of embryos with partial compaction patterns selected for transfer. In patients undergoing single blastocyst transfer without preimplantation genetic screening, identification of selection criteria that correlate to blastocyst implantation potential are essential. Further study of clinical outcomes with different compaction patterns is clearly needed.

In summary, this investigation applied time lapse microscopy for visualization of the entire compaction process. Compaction and morulation have been difficult to study in the past with once daily static observations. With TLM we were able to overcome these limitations. Partial compaction patterns in embryos correlated to delays in embryonic development, lower overall blastulation rates and poorer inner cell mass and trophectoderm quality when compared to embryos with full compaction. Additionally, blastocysts derived from partial compaction patterns were also more likely to be diagnosed as mosaic upon PGT testing. Taken together, these findings suggest that irregular compaction patterns with cell extrusion(s) and/or exclusion(s) are reflective of overall embryo quality and maybe potentially useful in embryo selection. To this end, a clinical trial with a larger study group is needed to determine if partial compaction should be considered as a negative feature when ranking blastocysts for transfer.

## Conclusions

Time-lapse visualization of compaction patterns identified exclusions and/or extrusions as negative indicators of blastocyst formation and blastocyst grade. When compared to full compaction patterns, partial compaction patterns were associated with delayed embryonic development as well as lower rates of optimal kinetic development.

## Data Availability

The datasets used and/or analyzed during the current study are available from the corresponding author on reasonable request.
