# Correction to: The presence of partial compaction patterns is associated with lower rates of blastocyst formation, sub‑optimal morphokinetic parameters and poorer morphologic grade

**DOI:** 10.1186/s12958-023-01117-2

**Published:** 2023-07-13

**Authors:** Christine Hur, Vaani Nanavaty, Meng Yao, Nina Desai

**Affiliations:** 1 Department of Obstetrics and Gynecology, Division of Reproductive Endocrinology and Infertility, Women’s Health Institute, Cleveland Clinic, 26900 Cedar Road, Beachwood, OH 44122 USA; 2 Quantitative Health Sciences, Cleveland Clinic, 9500 Euclid Ave. JJN3, Cleveland, OH 44195 USA

**Correction: Reprod Biol Endocrinol 21, 12 (2023).**

10.1186/s12958-023-01059-9

Following publication of the original article [1], the authors updated the corresponding e-mail from hurc@ccf.org to chur@IVF1.com.

The original article [1] has been updated.
